# Arthroscopic Excision for Intra-Articular Osteoid Osteoma of the Olecranon Fossa: A Case Report and Literature Review

**DOI:** 10.1155/2020/4034989

**Published:** 2020-07-06

**Authors:** Koichi Yano, Yasunori Kaneshiro, Hideki Sakanaka

**Affiliations:** Department of Orthopaedic Surgery, Seikeikai Hospital, 1-1-1 Minamiyasuicho, Sakai-ku, Sakai City, Osaka 590-0064, Japan

## Abstract

Intra-articular osteoid osteoma (OO) of the elbow is rare. We report a 26-year-old man who presented with pain, swelling, and limited elbow range of motion. Plain computed tomography (CT) showed a radiolucent round lesion at the distal humerus and reactive bone in the olecranon fossa. Conservative treatment with salicylate failed under the suspicion of OO. During elbow arthroscopy, a red solitary lesion was noted after resection of the white reactive bone in the olecranon fossa and was excised en bloc using a bony chisel. Histological examination showed OO. The patient's symptoms resolved the day after surgery. The patient remained asymptomatic 2 years postoperatively. This case report shows the successful clinical results of an arthroscopic procedure for intra-articular OO based on two primary goals: precise location of the lesion indicated by reactive bone on preoperative CT and histological verification using bony chisel.

## 1. Introduction

Osteoid osteoma (OO) is a type of benign bone tumor with a frequent incidence in the first to third decades. About 50% of OO cases occur in the diaphysis and metaphysis of the femur and tibia; the involvement of the elbow is rare and accounts for approximately 3% of OO [1, 2]. Additionally, intra-articular lesion accounts for approximately 10% of OO cases [3].

The typical symptom is pain that increases at night and can be reduced remarkably by salicylate or nonsteroidal anti-inflammatory drug (NSAID) pharmaceutical intervention. Other approaches for excision of the nidus for patients with pain or those unable to continue taking NSAIDs are surgical (wide resection or curettage) and percutaneous treatments [4, 5].

Open surgical resection may need internal fixation or bone graft due to risk of fracture. Recently, computed tomography- (CT-) guided radiofrequency ablation (RFA) has become a choice of treatment because of its minimal invasion, low cost, functional restriction, and high rate of pain reduction [5]. However, histological diagnosis could only be confirmed in 33–73% of patients, and RFA for treating intra-articular lesion may cause thermal damage to the articular cartilage [5–7]. Excision under arthroscopy for lesions located close to the neurovascular structures or intra- or juxta-articular lesions has recently become another treatment option [8].

In this report, we present a case of intra-articular OO of the olecranon fossa treated using arthroscopic resection with a chisel. A definite diagnosis was obtained histologically, and the literature was reviewed.

## 2. Case Presentation

Informed consent for publication of this case report was obtained from the patient, and this case report was approved by the institutional review board. A 26-year-old, right-handed man presented with right elbow pain without a history of trauma. His job involved desk work and had no relevant past medical history. One month after the onset of symptom, he visited an outpatient clinic where he was diagnosed with tendinitis and treated with physical therapy and NSAIDs. After 3 months, his symptoms had not improved, and he went to another hospital. He was suspected to have monoarthritis of the elbow and examined using blood test. Intra-articular steroid was injected to the elbow and physical therapy was also performed for 4 months, but they failed to improve his condition. He was referred to our hospital for further treatment at 11 months after the onset of symptoms.

**Figure 1:**
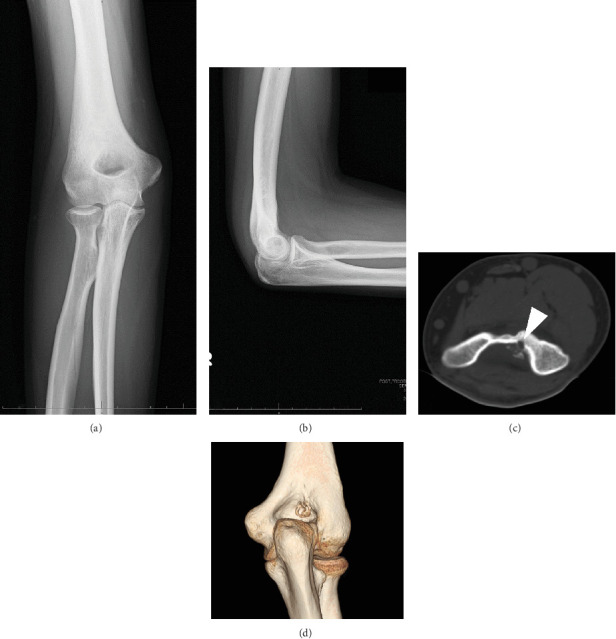
Preoperative plain radiography and computed tomography (CT). (a) Anteroposterior and (b) lateral views. No remarkable findings. (c) Axial and (d) three-dimensional CT images show the nidus (white arrowhead) in the distal humerus and reactive bone at the olecranon fossa.

**Figure 2:**
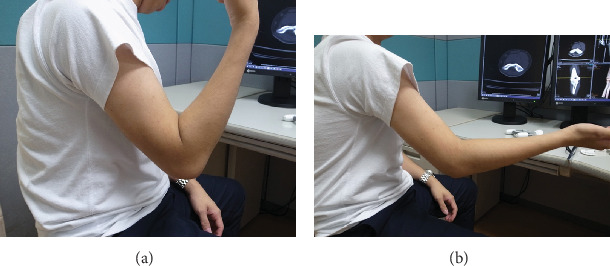
Preoperative photograph. (a) Elbow flexion and (b) extension.

Initial examination showed muscle atrophy of the right upper extremity and swelling of the right elbow. Neurovascular deficits were not observed. Range of the motion of the right elbow, measured using a standard goniometer, was 140° in flexion and -20° in extension. Plain radiography of the right elbow showed no remarkable finding (Figures 1(a) and 1(b)). Plain CT showed a radiolucent round lesion of 4 mm diameter at the distal humerus and reactive bone in the olecranon fossa (Figures 1(c) and 1(d)). The patient's chief complaint was persistent elbow pain, which increased with daily and working activities including writing letters and after use. OO was suspected on plain CT, and aspirin was prescribed after options for conservative and surgical treatments were explained to the patient. Positive effects of aspirin treatment on the symptoms were short-term, for a month, and the patient's ability to work was disturbed. He requested a surgical treatment. Preoperative examination showed that grip strength of the right and left hands, measured using a dynamometer, were 20.8 kg and 30.0 kg, respectively. The respective range of motion for the right and left extremities was as follows: elbow flexion, 115° and 145°; elbow extension, -35° and 5°; forearm pronation, 90° and 90°; and forearm supination, 90° and 90° (Figures 2(a) and 2(b)). The Mayo elbow performance score (MEPS) was 45, and the Disability of Arm, Shoulder, and Hand (DASH) score was 45 in disability and 75 in work.

**Figure 3:**
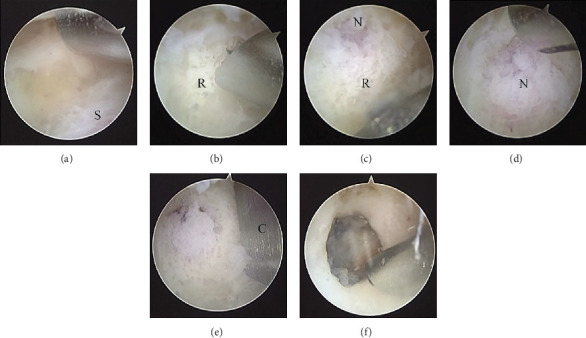
Photographs during arthroscopy. (a) Synovectomy. S: synovitis. (b) Resection of the reactive bone (R). (c) The nidus (N) under the reactive bone. (d) The nidus. (e) Resection of the nidus using a bone chisel (C). (f) Postresection of the nidus.

**Figure 4:**
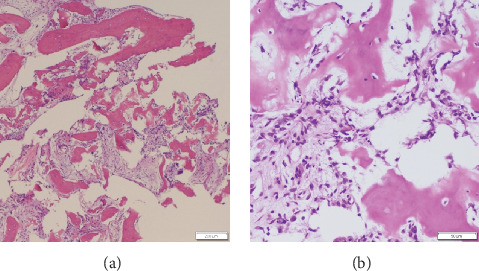
Histopathologic photograph. (a, b) Histological sections stained with hematoxylin and eosin show irregular osteoid and lining osteoblasts with scattered osteoclasts and significant vascular stromal tissue. Scale bar: 200 *μ*m (a) and 50 *μ*m (b).

**Figure 5:**
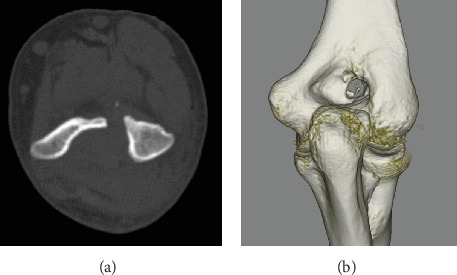
Postoperative computed tomography (CT). (a) Axial and (b) three-dimensional CT images show appropriate resection of the nidus.

Arthroscopy (4.0-mm diameter, 30° oblique arthroscope, Stryker K. K., Tokyo, Japan) with two portals (a direct posterior portal and a tumoral portal which was defined as the lateral corner of the olecranon fossa using a fluoroscope) was performed with the patient in the prone position using a pneumatic tourniquet under general anesthesia. Synovitis was filled in the joint, and it was resected for an appropriate surgical field using a shaver (Figure 3(a)). At the lateral side of the olecranon fossa, a red solitary lesion considered a tumor was noted after the resection of white reactive bony lesion (Figures 3(b)–3(d)). A bony chisel of 8 mm width was used to excise the tumor en bloc (Figure 3(e)). The excised specimen was sent for pathological examination. After excision, the cortical and cancellous bone around the lesion was resected until normal tissue was observed (Figure 3(f)). Histological examination showed OO (Figures 4(a) and 4(b)). Postoperative plain CT showed an appropriate resection of the nidus (Figures 5(a) and 5(b)).

**Figure 6:**
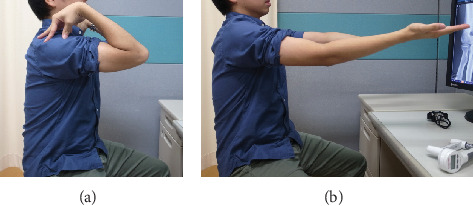
Postoperative photograph. (a) Elbow flexion and (b) extension 2 years postoperatively.

Preoperative pain resolved on postoperative day 1, and the patient was encouraged to use his extremity freely. At the final follow-up, 2 years postoperatively, he was asymptomatic. Grip strength was 33.2 kg and 30.9 kg for the right and left hands, respectively. Range of motion of the right elbow was as follows: flexion, 135°; extension, -15° (Figures 6(a) and 6(b)). MEPS was 85, and the DASH score was 1.7 in disability and 0 in work.

## 3. Discussion

The present case showed various symptoms, and 11 months was necessary to diagnose OO using CT after the onset of symptoms. Arthroscopic surgery was performed on the reactive bone in the olecranon fossa as a landmark for tumor. A nidus following resection of the reactive bone was resected en bloc using a bone chisel, and histological verification was performed.

Patients with typical OOs in the cortex of diaphysis of the long bones present with nocturnal pain alleviated by NSAID treatment as a symptom and a radiolucent nidus with surrounding reactive sclerosis or cortical thickening as a radiographic finding [9]. Conversely, intra-articular OOs have atypical symptoms including pain, synovitis, limited range of motion, joint effusion, and contracture and little or no reactive sclerosis because the periosteum is absent as a radiographic finding [10, 11]. Moreover, nocturnal pain is absent, and the pain is less responsive to NSAID treatment [2]. Intra-articular lesion of OO in the elbow is rare and, therefore, can be misdiagnosed as tenosynovitis, monoarthritis, tendinitis, and chondral lesions [1, 12]. Szendroi et al. reported that the average time from presentation to diagnosis for intra-articular lesions was 26.6 months and that for other lesions was 8.5 months [13].

Thin-section and multiplanar reconstruction CT is recommended as a preoperative (diagnostic and localization) and postoperative (appropriate excision) examination and was also useful preoperatively and postoperatively in our case [1, 14].

Complete resection of the lesion is mandatory for the surgical treatment of OO [15]. Incomplete resection causes incomplete resolution of symptoms or recurrent symptoms. Pain relief can be achieved independent of any type of intervention. The clinical success necessitates the precise identification of the lesion.

Regarding open surgical resection, a posterior approach without olecranon osteotomy or with triceps reflexion was performed for a similar case with OO of the olecranon fossa [16, 17]. However, opening the joint capsule or excising the muscle requires a long time for the recovery of function in the patient.

In CT-guided RFA, RFA is performed after bony biopsy, and the size of the nidus is small (<2 cm) [18]. Thus, we suppose histological verification is not completely accurate (from 36 to 73%) and obtaining a specimen might require special skills [5, 6].

Although some authors stated that clinical and radiological findings are valuable to diagnose OOs, we believe that a definite diagnosis should be obtained by histology because it is a space-occupied lesion [4, 5].

For intra-articular lesions, arthroscopic-assisted resection is also a treatment option. The advantage of arthroscopy is reduced postoperative pain related to minimal incisions, fewer wound problems, wider intraoperative vision, a less invasive surgery, and earlier return to full activity levels [13]. To the best of our knowledge, 19 cases in 1 case series and 8 case reports describe arthroscopic resection for OO in the elbow [8, 19–26]. Six patients with OO in the olecranon fossa are among these cases. Two cases were re-operated, one had incomplete removal of the anterior trochlea, and the other had unaccessible tumor of the radial head [8, 19]. Histological verification for OO was obtained in 10 of 17 cases (no description available in 2 cases). Kamani et al. reported that ablation with the burr was used for cases with no visible lesion, and this procedure might reduce the specimen into fragments and histological confirmation was obtained in only 4 of 10 cases [8]. Thus, some authors described that it was difficult to obtain a histological assessment in the arthroscopic removal of the nidus [8, 20, 22]. On the contrary, we could perform histological confirmation, and we recommend the use of a bone chisel for en bloc resection when possible.

Regarding the precise location of the OO, a reactive bone was noted in the olecranon fossa on preoperative CT in our case. The nidus of OO generally accompanies a surrounding sclerotic bone when the nidus localizes in a cortical or cancellous bone. Although the intra-articular lesion has little or no reactive sclerotic bone, some cases, as in our case, have reactive bone in the articular surface. This reactive bone provides guidance to the nidus location because it indicates that the nidus exists under the reactive bone.
